# The prognostic significance of single‐nucleotide polymorphism array‐based whole‐genome analysis and uniparental disomy in myelodysplastic syndrome

**DOI:** 10.1111/ijlh.13502

**Published:** 2021-03-02

**Authors:** Yang Ou, Yan Yang, Hongbin Yu, Xin Zhang, Min Liu, Yu Wu

**Affiliations:** ^1^ Department of Hematology and Hematology Research Laboratory West China Hospital Sichuan University Chengdu China

**Keywords:** metaphase cytogenetic analysis, myelodysplastic syndrome, uniparental disomy

## Abstract

**Introduction:**

Myelodysplastic syndrome (MDS) is a group of heterogeneous hematological diseases characterized by ineffective hematopoiesis and dysplastic morphology. Single nucleotide polymorphism array (SNP‐A)‐based whole genome analysis has a much higher resolution for chromosomal alterations when compared with conventional cytogenetic tools. In the present study, we evaluated the diagnostic value and prognostic significance of SNP‐A in MDS patients with normal karyotypes.

**Methods:**

A total of 127 patients with MDS and myeloproliferative neoplasms or acute myeloid leukemia with myelodysplasia‐related changes were included in our study. The advantages and disadvantages of SNP‐A were compared with those of traditional metaphase cytogenetic analysis (MC). The Kaplan‐Meier analysis and COX regression analysis were used to investigate the prognostic value of SNP‐A and uniparental disomy (UPD) in MDS patients with normal karyotype. Furthermore, the chromosomal abnormalities detected by SNP‐A in patients with specific gene mutations were explored.

**Results:**

SNP‐A was more sensitive toward meaningful chromosomal aberrations (58.2% vs 36.9%; *P* < .05) than MC. Among the patients with normal karyotype, those who were detected with new chromosomal abnormalities via SNP‐A presented with inferior survival compared with those without the abnormalities (*P* = .003). Additionally, the presence of UPD was an independent prognostic factor in patients with normal karyotype (*P* = .01). TP53 and RUNX1 mutations often occurred with abnormalities in chromosomes 17p and 21q, respectively.

**Conclusions:**

Compared with MC, SNP‐A capable of detecting UPD can offer more diagnostic and prognostic information; TP53 and RUNX1 gene mutations are often accompanied by abnormalities in their chromosomes (17p, 22q).

## 1 INTRODUCTION

Myelodysplastic syndrome (MDS) is a group of hematological diseases characterized by ineffective hematopoiesis and a dysplastic morphology; some patients with excessive marrow blasts may present with acute myeloid leukemia (AML).^1^ MDS is a heterogeneous type of disease, that is, the course of the disease may vary in the patient based on their clinic‐pathological features (including age and history of chemotherapy, among others).

Besides the blast percentage, patients with MDS may be classified into different risk groups using prognosis scoring systems. The International Prognostic Scoring System (IPSS) and the revised IPSS (IPSS‐R) have been most commonly used to evaluate the prognosis of patients with MDS, and the cytogenetic index occupies a significant position in these systems.^2^, ^3^ However, the clinical manifestations of this disease may differ among the patients in the low‐risk and intermediate‐1 (int‐1) groups when evaluated by IPSS or IPSS‐R. Some low‐risk patients may present with a progressive course, whereas several patients in the int‐1 group may have a better prognosis. Several other important scoring systems for MDS, including the MD Anderson Cancer Center MDS model, mainly target patients belonging to the low‐risk and int‐1 groups but do not yield idealistic effects.^4^

Nearly half (50%) of patients with MDS are known to present with chromosomal abnormalities.^5^, ^6^ Cytogenetic alteration has been determined as an essential prognostic factor in these patients. Conventional cytogenetic tools include metaphase cytogenetic analysis (MC) and fluorescence in situ hybridization (FISH). A routine MC can be used to examine numeric and structural alterations in the chromosomes at the single‐cell level. Despite its low sensitivity and the need for technical proficiency, MC has a remarkable advantage in detecting novel cytogenetic abnormalities.^7^ Additionally, FISH can be used to detect numeric and structural alterations in the chromosomes, but it cannot discover novel alterations, despite its superior sensitivity and ease of handling. Thus, MC remains as the main tool used to examine alterations in the chromosomes, whereas FISH is used as a supplement for sensitivity.

Single nucleotide polymorphism array (SNP‐A)‐based genome‐wide analysis technology can be used to examine the imbalanced alterations of somatic or clonal cells in hematopoietic diseases.^1^ This method has a much higher resolution when compared with conventional cytogenetic tools and has the advantage of discovering unknown potential alterations when compared with FISH.^8^, ^9^ Additionally, SNP‐A could detect uniparental disomy (UPD), which could not be detected by MC or FISH.^1^ UPD refers to a pair of homologous chromosomes in which one (paternal or maternal chromosome) is duplicated and the opposite one is deleted.^10^ Primary UPD is associated with errors in meiosis and may lead to some growth‐ and development‐related diseases.^11^ Acquired UPD, such as other chromosomal abnormalities, can be used as a marker of cloning in malignant tumors.^12^ An increasing number of studies have found that cancer cells may gain clonal advantages through acquired UPD, such as homozygous mutation of a part of the JAK2 V617F gene associated with UPD 9p.^13^, ^14^ Some gene mutations often occur with abnormalities of the chromosome in which they are located.^15^ However, the prognostic significance of the presence of UPD in hematologic malignant diseases remains to be determined. Additionally, the association between chromosome abnormalities and molecular genetics should be examined.

In the present study, we evaluated the cytogenetic characteristics of MDS patients, compared the detection yields of the chromosome aberrations between the SNP‐A and conventional cytogenetic examinations, and determined the prognostic significance of SNP‐A and UPD.

## 2 MATERIALS AND METHODS

### 2.1 Patient samples

Patients with MDS and related diseases, including myelodysplastic/myeloproliferative neoplasm (MDS/MPN) and AML transformed from MDS, who visited a tertiary hospital (West China Hospital of Sichuan University) from 2013 to 2019 were screened. The patients provided written informed consent, and the Ethics Committee of the West China Hospital of Sichuan University approved the study protocol.

The inclusion criteria for the patients in the present study were as follows: All patients should meet the standards of diagnosis in the 2016 revision of the World Health Organization classification of myeloid neoplasms and acute leukemia^16^; AML patients must have a preceding history of MDS; and all patients should have reliable SNP‐A results. Those who underwent chemotherapy for other cancers and were diagnosed with secondary myeloid neoplasms or acute leukemia were excluded from the present study.

### 2.2 Data collection and follow‐up

The patients were selected using the Hospital Information System (HIS), and general clinical data, including gender, age, and time of onset of the disease, were collected. The Laboratory Information System was used to obtain the results of the complete blood counts (including the hemoglobin, platelet count, white blood cell count, neutrophil percentage, and peripheral blood blast cell percentage) and bone marrow smear image analysis (marrow blast cell percentage and presence of myelodysplasia). The MC, FISH, SNP‐A, and myeloid neoplasm‐related gene mutation examinations were conducted by second‐generation sequencing. The clinical data were graded according to IPSS and IPSS‐R, and the survival conditions were followed up via telephone calls.

### 2.3 Cytogenetic and genetic examinations

The G‐banding technique was used for karyotype analysis (banding level, 200‐300 bands). Six sets of probes (D5S23, D5S721/CSF1R, D7Z1/D7S486, D8Z2, D20S108, DXZ1/DYZ1, and p53/CEP17) were used to detect abnormalities in the 5p12.2/5P33‐34, 7p11.1‐q11.7q31, 8p11.1‐q11.1, 20Q12, and Xp11.1‐Q11.1/Yq12, 17p13.1/17p11.1‐q11.1 chromosomes via FISH. CytoScan 750K Assay and CytoScan 750K Array‐Affymetrix were used for the genome‐wide detection of chromosomal imbalance aberrations via SNP‐A; the entire process was conducted strictly according to the quality control criteria. The CytoScan 750K chip has more than 750 000 probes for copy number variation analysis (comprising 550 000 unique, nonpolymorphic probes and approximately 200 000 SNP probes with high accuracy) and covers 4127 genes. To alleviate the false‐positive rate, thresholds of Gain ≥ 400 Kb, Loss ≥ 400 Kb, and UPD ≥ 5 Mb, the number of probes ≥ 50 are set for significant chromosomal abnormalities. Chromosomal abnormalities above these thresholds are reported, and chromosomal abnormalities below this threshold are not reported unless they can be verified by other detection techniques such as NGS, FISH, and MLPA, and there is evidence that they are linked with hematological malignancies at the same time. Second‐generation sequencing was used during molecular genetic examination to detect the most common mutation sites in genes, such as TET2, TP53, SF3B1, ASXL1, and RUNX1, in patients with myeloid neoplasms.

### 2.4 Statistical analysis

The R language (Bell Laboratories; Lucent Technologies) was used for statistical analysis. The chi‐square test and Fisher's exact probability method were used to compare the merits and demerits of SNP‐A and the conventional method of cytogenetic examination. The Kaplan‐Meier (KM) method and COX regression analysis were used (“survival” package^17^) to analyze the overall survival (OS) of the patients in the different subgroups, classified according to the cytogenetic results and to draw the survival curves. Chi‐square and Fisher's exact probability tests were used to test the independence of the chromosomal abnormalities and hot‐spot gene mutations. The “karyoploteR” package was used to map the genomic alterations.^18^ Hypothesis testing was statistically significant when *P* < .05.

## 3 RESULTS

### 3.1 General clinical information

**FIGURE 1:**
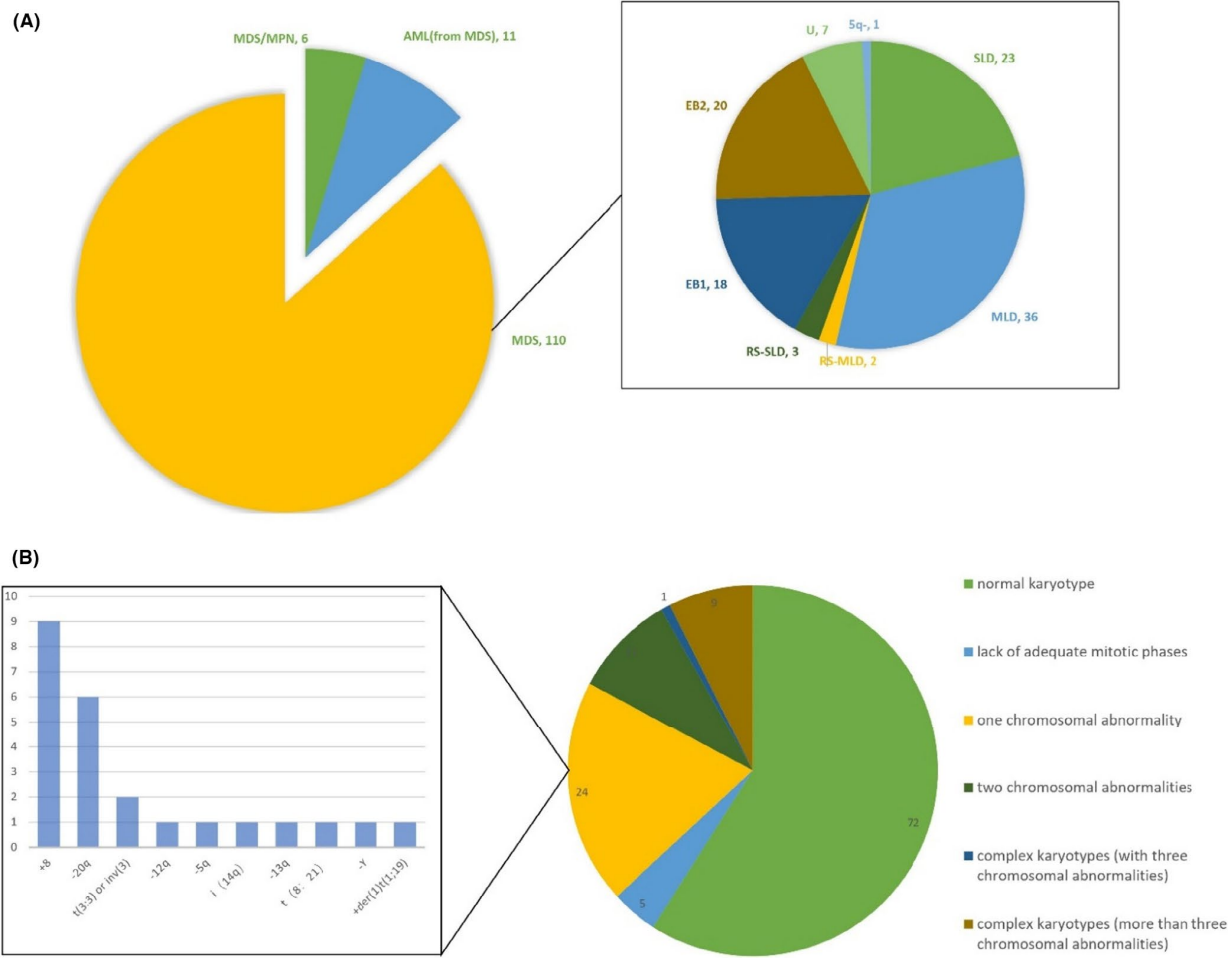
The diagnostic information of patients included in the present study: A, The diagnostic composition of all 127 patients and the classification of all 110 patients with MDS. B, MC results of patients included in the present study. SLD: MDS‐SLD, myelodysplastic syndromes with single lineage dysplasia; MLD: MDS‐MLD, myelodysplastic syndromes with multilineage dysplasia; RS‐SLD: MDS‐RS‐SLD, myelodysplastic syndrome with ring sideroblasts with single lineage dysplasia; RS‐MLD: MDS‐RS‐MLD, myelodysplastic syndrome with ring sideroblasts with multiple lineage dysplasia; EB1: MDS‐EB‐1, myelodysplastic syndromes with single lineage dysplasia with excess blasts 1; EB2: MDS‐EB‐2, myelodysplastic syndromes with single lineage dysplasia with excess blasts 2; U: MDS‐U, myelodysplastic syndromes unclassifiable; 5q‐: myelodysplastic syndrome with isolated del(5q)

Based on the inclusion and exclusion criteria, 127 patients—including 110 patients diagnosed with MDS (Figure 1A)—were analyzed. Patients' clinical characteristics are shown in Supplementary 1.

### 3.2 Cytogenetic and genetic examinations

In the present study, one hundred and twenty‐two patients completed MC, whereas sufficient cytogenetic results were not obtained from five patients because of the lack of the mitotic phase, and detailed MC results are shown in Figure 1B. Only 10 patients underwent FISH examination, and abnormal signals of 8p11‐q11 were detected in two patients, whereas the rest were negative.

**FIGURE 2:**
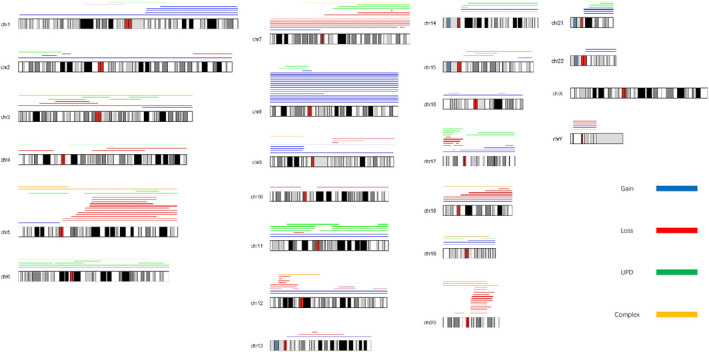
Summary of genomic alterations detected by SNP‐A. Gain: duplication or duplicated alteration; Loss: deletion or deleted alteration; UPD: uniparental disomy; and Complex: complex changes or chromothripsis)

Using SNP‐A, 206 genomic changes, including 43 UPDs, 57 duplications, 93 deletions, and 13 complex changes (representing chromothripsis), were observed in all 127 patients (Figure 2).

Of the 71 patients who underwent molecular genetic examinations, 53 presented with 95 meaningful mutations. The most commonly mutated gene was TET2 (16 patients; 22.5%) followed by TP53 (11 patients, 16.4%), SF3B1 (10 patients, 14.9%), ASXL1 (nine patients, 12.7%), and RUNX1 (seven patients, 10.4%).

### 3.3 Merits and demerits of SNP‐A compared with MC

The results of SNP‐A and MC were improved in 122 patients. As shown in Table 1, the positivity of SNP‐A for significant chromosomal aberrations was higher than that observed with the MC (58.2% vs 36.9%; *P* < .05). Increased and specific information about the chromosomal abnormalities was obtained via SNP‐A when compared with the MC method in 10 patients with complex karyotypes (no less than three chromosomal aberrations detected by MC). Besides complex karyotypes, SNP‐A found 78 chromosomal alterations (38 UPDs and 33 mosaic deletions or duplications) that were not detected by MC in 40 patients. Six balanced translocations were detected by MC, whereas SNP‐A was negative due to invariable copy number. Additionally, MC detected two marked chromosomes in the two patients, which needed further verification. MC combined with SNP‐A significantly improved the diagnostic efficiency of chromosome defects when compared with MC alone (61.4% vs 37.5%; *P* <.05).

**TABLE 1:** Positive numbers and rates of chromosomal defects between the SNP‐A and MC techniques

		MC		
		Positive	Negative	Overall
SNP‐A	Positive	41 (33.6%)	30 (24.6%)	71 (58.2%)
	Negative	4 (3.3%)	47 (38.5%)	51 (41.8%)
	Overall	45 (36.9%)	77 (63.1%)	122 (100%)

Abbreviations: MC, metaphase cytogenetic analysis; SNP‐A, single nucleotide polymorphism array (SNP‐A)‐based genome‐wide analysis.

### 3.4 Prognostic significance of SNP‐A and UPD

The effect of the newly discovered abnormalities via SNP‐A on the prognosis was further analyzed in 63 MDS patients. Among them, no new chromosomal abnormalities were detected by SNP‐A in 41 patients, whereas 22 patients presented with new abnormalities. These patients with new abnormalities could be categorized into two groups: 15 had UPDs, which were negative in MC, and the remaining seven had mosaic deletions or duplicates, which indicated that the structural alterations occurred in a proportion but not all of the examined cells. The time of onset was retrieved from the HIS system, and the patients were followed up via telephone calls. Of the 63 patients, 43 survived, 10 died, and 10 were lost to follow‐up.

**FIGURE 3:**
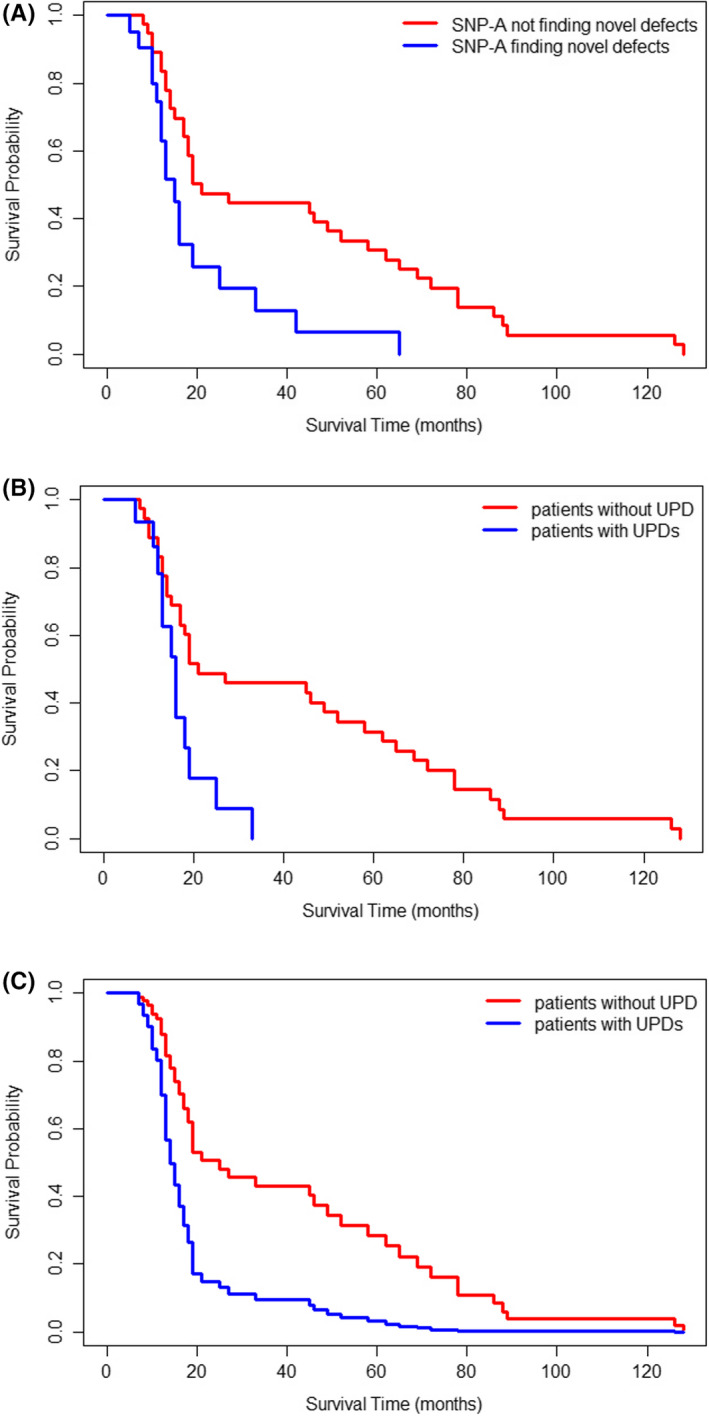
Overall survival (OS) of MDS patients with normal karyotype. A, Survival curves in 41 patients without novel chromosomal defects found by SNP‐A (red line) and 22 patients with novel chromosomal defects found by SNP‐A (blue line) using the KM method (*P* = .003). B, Survival curves in 41 patients without chromosomal structural abnormalities or UPDs (red line) and 15 patients with UPDs (blue line) using the KM method (*P* = .008). C, Survival curves in 41 patients without chromosomal structural abnormalities or UPDs (red line) and 15 patients with UPDs (blue line) using the COX multivariate regression model (adjusted for the average age, *P* = .01)

The 63 patients with normal karyotype detected by MC were divided into an SNP‐A positive (22 cases) and SNP negative (41 cases) group. The KM method was used to analyze the survival of the MDS patients with normal karyotype; patients with new chromosomal abnormalities found by SNP‐A had a worse prognosis than those without new abnormalities (Figure 3A; *P* = .003, median survival time: 15 vs 21 months). After excluding all patients with structural alterations (abnormal karyotypes detected by MC and mosaic deletions found by SNP) and comparing the survival probabilities of 41 patients without SNP‐A and 15 patients with UPD, we found that those with UPD tended to have a poorer prognosis than those without UPD (Figure 3B; *P* = .008, median survival time: 16 vs 21 months). However, the survival curve demonstrated overlapping indicating that there may be other factors that could affect the prognosis of the patients. Therefore, the COX univariate analysis was utilized to analyze the overall survival using factors such as age, platelet count, neutrophil count, hemoglobin, blasts percentage, IPSS‐R, and the presence or absence of UPD. Age (*P* = .02) and the presence of UPD (*P* = .01) were determined as the independent prognostic factors (Supplementary 2). Based on these two variables, a COX multivariate regression model (likelihood ratio test *P* = .003) was constructed, and the survival curves were created after adjusting for age (Figure 3C). Patients with UPD had a worse prognosis than those without UPD (*P* = .01).

### 3.5 Chromosomal alterations and gene mutations

**FIGURE 4:**
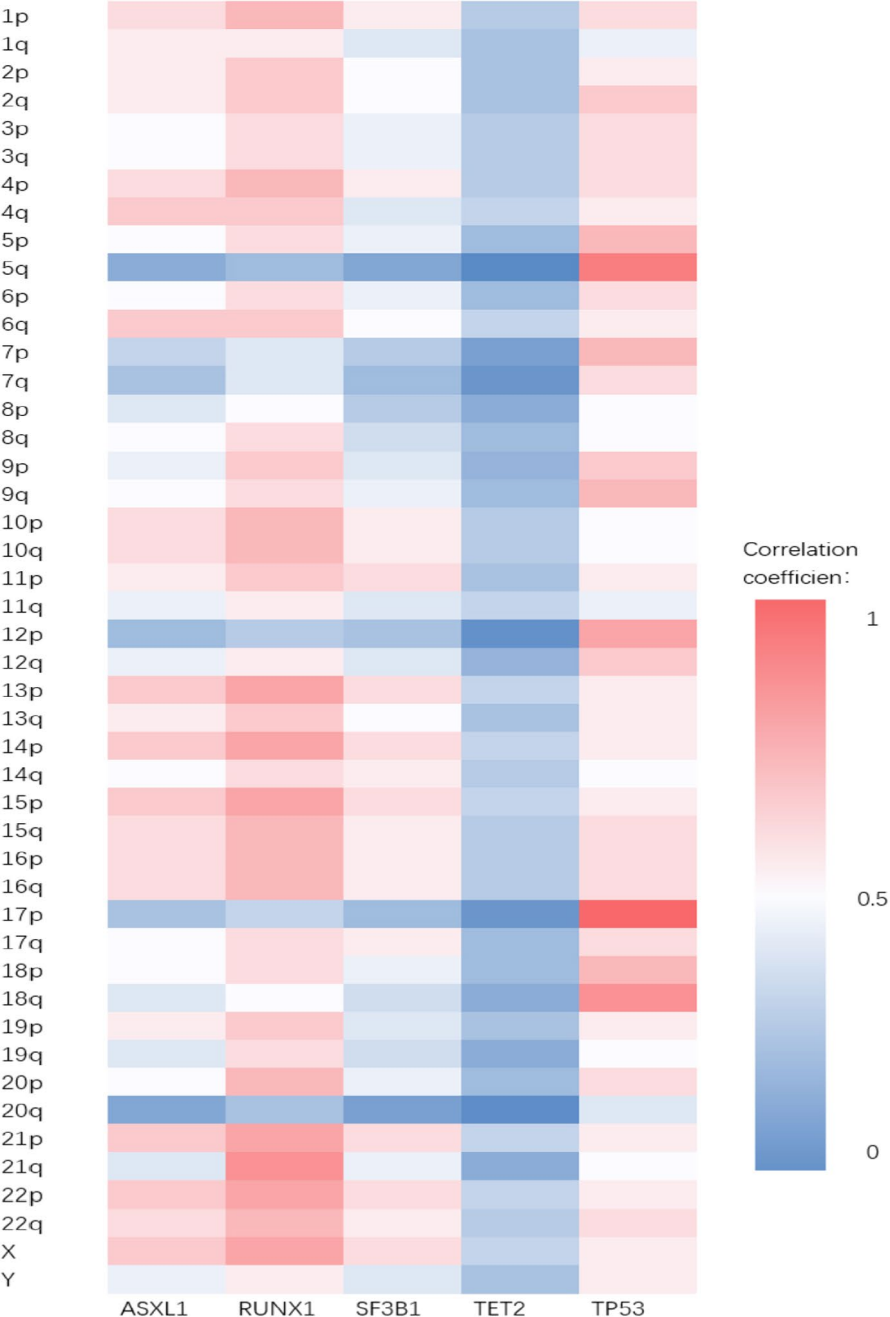
The heatmap of the correlation between significant gene mutations and genomic alterations detected by SNP‐A

Correlations between the chromosomal abnormalities detected by SNP‐A and the different gene mutations (including ASXL1, RUNX1, SF3B1, TET2, and TP53) were analyzed, and a heatmap was constructed (Figure 4). TP53 mutation was often accompanied by abnormalities in the chromosome where it was located (17P; *P* < .05). Among the 11 patients with both TP53 mutation and 17P alteration, four had duplications, three had UPD, and one had deletions. RUNX1 gene mutations were also associated with their corresponding chromosomal (22q) abnormalities (*P* < .05); all abnormalities in chromosome 22q were UPDs. No other correlations were observed between the chromosomal abnormalities and the gene mutations.

## 4 DISCUSSION

Myelodysplastic syndrome patients are highly heterogeneous and present with varying clinical manifestations, prognosis, and risks of transformation to AML. Most researchers have focused on the molecular genetics, such as the TP53 gene^19^, ^20^, ^21^, ^22^ (which is closely related to complex karyotypes and an increased risk of progressing to leukemia) and important DNA methylation genes, such as TET2 and DNMT3a.^23^ In the present study, the SNP‐A results of 127 patients with MDS and MDS‐related diseases (MDS, 110; MDS/MPN, 6; and transformed AML, 11) were compared with those obtained via MC. The clinical data, results of the genetic tests, and survival were analyzed to determine the prognostic significance of SNP‐A and UPD in MDS.

### 4.1 Compared with the MC, the resolution of SNP‐A is superior to detect significant chromosome aberrations

The SNP array is designed using a comparator DNA, RNA, or tissue that is arrayed on a glass slide or glass beads, instead of a normal human control.^24^, ^25^ It covers the entire genome with an average resolution of approximately 35 kb throughout the genome. The typical resolution of MC is about 400 bands; a single chromosome band may contain 6 megabases of DNA and approximately 150 genes. The advantages of the resolution of SNP‐A are more apparent in MDS patients, especially those without adequate mitotic phases due to myelofibrosis and hypoplasia. Additionally, SNP‐A is superior to MC in its ability to identify loss of heterozygosity (LOH) or acquired UPD. The prognostic significance and pathogenic role of acquired UPD are being increasingly recognized in MDS and AML.

Tiu et al^27^ reported that the combination of MC and SNP‐A could greatly improve the detection rate of chromosomal defects (74% vs 44%; *P* < .0001). The detection rate in the present study was lower than that reported by Tiu et al because of the lower proportion of patients with AML. There were some other researches investigating the diagnostic value and prognostic significance of SNP‐A in MDS patients with normal karyotypes,^7^, ^26^, ^28^ which may interpret the clinical variability, and our research has given a deeper insight into the interpretation of SNP‐A's results.

### 4.2 Patients with additional abnormalities found by SNP‐A or UPDs had a worse prognosis than those without

Tiu et al analyzed the survival data of 430 cases of MDS and MDS‐related diseases and found that regardless of the MC results, the discovery of new chromosomal defects through SNP‐A predicted a worse prognosis; moreover, the higher the number of new defects detected, the poorer the prognosis.^27^ However, to the best of our knowledge, there is no published research on the prognostic significance of UPD. In the present study, 63 patients with normal karyotypes were selected for further analysis. Patients with new chromosomal abnormalities found by SNP‐A had a lower survival probability than those without new chromosomal abnormalities (*P* = .003). Additionally, the presence of UPD was an independent prognostic factor in patients with normal karyotypes (*P* = .01). Furthermore, only seven out of the 63 normal karyotype patients belonged to the very‐high‐risk group in the IPSS‐R. Notably, in the single‐factor COX regression analysis, variables like platelet count, absolute neutrophil count, hemoglobin count, the proportion of the blasts, and IPSS‐R scores could not predict prognosis, while whether with UPD and age had prognostic significance. It is worthwhile to investigate the role of UPD in the stratification of relatively lower‐risk MDS patients.

### 4.3 TP53 and RUNX1 gene mutations are often accompanied by abnormalities in the chromosomal regions where they are located (17p and 22q)

TP53 gene mutations often occurred along with abnormalities in the chromosome (17p) where they were located (*P* < .05). Similarly, RUNX1 gene mutations were observed along with abnormalities in their chromosomal locations (22q; *P* < .05). These findings are similar to those reported by Jasek et al.^15^ To some extent, it proved the validity of the “double hit” (or “multi‐hit”) theory ^29^; those patients may have a heterozygous TP53 gene, after the loss of one of homologous 17p chromosomes (meanwhile loss of wild‐type allele), the TP53 gene goes inactivation.^30^ LOH of 17p may occur because the homologous chromosome of the mutant is replicated to correct the missing copy number. A recent study found that the TP53 multi‐hit state predicted the risk of death and leukemic transformation independent of the IPSS‐R method, and monoallelic patients did not differ from the TP53 wild‐type patients in terms of the outcomes and response to therapy.^31^

Although many prognostic models have been used in patients with MDS, the pathophysiological mechanism of this condition involves multiple aspects and steps, and the corresponding treatments are currently being evaluated. In the present study, from the cytogenetics viewpoint, we explored the prognostic effect of SNP‐A and UPD in MDS and provided a new perspective for risk stratification.

## CONFLICT OF INTEREST

The researchers claim no conflicts of interest.

## Supporting information

Supplementary Table 1

Supplementary Table 2

## Data Availability

Author elects to not share data.
