# Supplementary material for: The prognostic significance of single‐nucleotide polymorphism array‐based whole‐genome analysis and uniparental disomy in myelodysplastic syndrome
Source: Int J Lab Hematol. 2021 Mar 2;43(5):1062–9. doi: 10.1111/ijlh.13502 (PMC8518839; doi:10.1111/ijlh.13502)
Supplement: Supplementary file 1 — Supplementary Table 1 [file IJLH-43-1062-s002.docx]

**Supplementary 1.** General clinical information and hematological indices in the 127 patients included in the present study: The MDS patients had an average age of 53.7 years. The mean hemoglobin of MDS patients was 78.2g/L, the mean white blood cell count was 3.9×10^9/L, the mean platelet count was 98.3×10^9/L, the mean neutrophil percentage was 48.3%, and the mean blast cell percentage of bone marrow smear was 4.2%.

|  | MDS | MDS/MPN | AML  (with MDS changes) | Overall |
| --- | --- | --- | --- | --- |
| Number | 110 | 6 | 11 | 127 |
| Gender（male/female） | 63/57 | 5/1 | 6/5 | 74/63 |
| Age | 53.7 ± 16.0 | 58.5 ± 19.5 | 63.3 ± 10.0 | 54.7 ± 16.0 |
| Hemoglobin (g/L) | 78.2 ± 26.0 | 59.3 ± 14.1 | 83.5 ± 22.3 | 77.8 ± 25.6 |
| White blood cell count  (×10^9^/L) | 3.9 ± 3.4 | 55.6 ± 82.3 | 5.4 ± 5.6 | 6.5 ± 21.3 |
| Absolute neutrophil count  (ANC, ×10^9^/L) | 48.3 ± 18.2 | 52.7 ± 21.1 | 43.5 ± 20.1 | 48.1 ± 18.6 |
| Plate count  (×10^9^/L) | 98.3 ± 113.0 | 182.5 ± 4261.2 | 144.1 ± 155.2 | 106.3 ± 129.7 |
| Blast percentage (%) | 4.2 ± 4.8 | 2.5 ± 3.4 | 25.6 ± 5.8 | 6.1 ± 7.8 |

MDS: myelodysplastic syndrome; MPN: Myeloproliferative neoplasm; AML: acute myelocytic leukemia.
