# Supplementary material for: The prognostic significance of single‐nucleotide polymorphism array‐based whole‐genome analysis and uniparental disomy in myelodysplastic syndrome
Source: Int J Lab Hematol. 2021 Mar 2;43(5):1062–9. doi: 10.1111/ijlh.13502 (PMC8518839; doi:10.1111/ijlh.13502)
Supplement: Supplementary file 2 — Supplementary Table 2 [file IJLH-43-1062-s001.docx]

**Supplementary 2.** COX univariate prognostic analysis of 56 MDS patients without chromosomal structural abnormalities: age (P = 0.02), and whether UPD (P = 0.01) were independent prognostic factors, while variables like platelet count, absolute neutrophil count, hemoglobin count, the proportion of the blasts, IPSS-R scores could not predict prognosis.

|  | HR | CI | P-value |
| --- | --- | --- | --- |
| Presence of UPD | 6.37 | (1.297, 5.742) | 0.01 |
| Age | 5.20 | (1.049, 1.003) | 0.02 |
| Plate count | 0.03 | (0.997, 1.004) | 0.70 |
| Neutrophil count | 0.05 | (0.824, 1.264) | 0.83 |
| Hemoglobin | 0.15 | (0.984, 1.011) | 0.86 |
| Blast percentage | 0.97 | (0.973, 1.091) | 0.32 |
| IPSS-R scores | 0.59 | (0.7171, 1.159) | 0.44 |

HR: hazard rate; CI: confidence interval; UPD: uniparental disomy; IPSS-R: Revised International Prognostic Scoring System.
